# Factors That Influence Resilience among First-Year Undergraduate Nursing Students: A Cross-Sectional Descriptive Study

**DOI:** 10.3390/nursrep14020100

**Published:** 2024-05-24

**Authors:** Gopolang Gause, Leepile Alfred Sehularo, Molekodi Jacob Matsipane

**Affiliations:** 1 NuMIQ Research Focus Area, Faculty of Health Sciences, North-West University, Potchefstroom 2531, South Africa; molekodi.matsipane@nwu.ac.za; 2 Lifestyle Diseases Research Focus Area, Faculty of Health Sciences, North-West University, Mafikeng 2745, South Africa; leepile.sehularo@nwu.ac.za

**Keywords:** cross-sectional descriptive design, nursing students, resilience, undergraduate, South Africa

## Abstract

During their transition from basic to higher education, first-year undergraduate nursing students need to balance theoretical and clinical requirements, as well as their social life. A significant number of them struggle with this, due to a lack of coping mechanisms due to poor resilience. This study aimed to determine factors that influence resilience among first-year undergraduate nursing students at a South African university. A cross-sectional descriptive research design was followed, with stratified convenient sampling. Data were collected during August 2023 using an adapted self-administered online questionnaire. The reliability and validity of the adapted version was ensured in the context of this study. Principal component analysis and varimax rotation were used to analyse data. A total of 123 participants (47.2% from campus A and 52.8% from campus B) completed the questionnaire. The majority (88%) were females. This study showed that resilience can be dependent on various factors, such as lecturer support, parental support, academic achievement, peer and mentor support, optimism about the future, and self-determination. It is recommended that higher education institutions should consider incorporating the factors presented in this study as part of the broader orientation of first-year undergraduate nursing students when they first arrive at university.

## 1. Introduction

The transitioning period from basic to higher education can be problematic due to the variance between these two levels of education [1]. For first-year undergraduate nursing students in particular, this period is regarded as one of ‘juggling many balls’, because students need to balance the theoretical and clinical requirements, including maintaining a social life [1,2,3]. A significant number of first-year undergraduate nursing students succumb to these challenges by dropping out of the academic program, abusing drugs and the like, due to poor coping mechanisms. A study conducted by Park and Hong [4] suggests that the demanding expectations that first-year undergraduate nursing students experience often lead to a lack of academic confidence, which has an indirect influence on their adjustment and resilience. It is therefore important to understand the factors influencing resilience among first-year undergraduate nursing students [1]. 

The word ‘resilience’ is a derivative from the Latin word *resilia* which means the action of rebounding [5]. Within the empirical referents of many spheres of livelihood, the concept of resilience is better understood as the ability to bounce back when faced with adversity of any sort [2]. In nursing education, this can be equated to the ability to navigate through all factors which may negatively influence resilience during the course of training, until completion of the nursing program. However, there is an ongoing debate on whether resilience among first-year undergraduate nursing students is a psychological trait, an ability of some sort, or a process acquired over time [6]. Whatever its nature, the importance of resilience among first-year undergraduate nursing students cannot be over-emphasized.

According to Aryuwat et al. [6] as well as Pryjmachuk et al. [7], resilience is determined by several factors related to the demographic background of nursing students, which can either amplify or condense their resilience at the university. In addition, literature has shown that factors such as vocal behaviour, social support, creativity, immersion in senior students, and creative problem-solving skills have a positive influence on the adjustment to university life among first-year nursing students [4,8]. Similar findings were revealed by Haktanir et al. [1], who highlighted a positive correlation between resilience and nursing students’ attributes, such as high self-confidence, high self-compassion, high self-efficacy, and being positive. A study conducted in an urban university in South Africa among first-year undergraduate nursing students highlighted spirituality, exercise, and positive framing as buffers to their poor resilience [9]. However, a gap identified in the available literature is that an overwhelming majority of these studies were conducted either during or pre-COVID-19, and do not necessarily incorporate the post-COVID-19 era. Furthermore, there is a dearth of literature on studies exploring resilience among first-year undergraduate nursing students at predominantly rural universities like the university where this study was conducted. This is despite evidence showing that poor resilience and lack of coping with transition from basic to higher education is more prevalent on students from less affluent families [10].

While an acknowledgement is made of the universities’ attempt to smooth the transitioning process through orientation programs which provide an overall framework for success at the university [7,8], there is still a gap regarding the applicability of these programs to nursing students. Furthermore, there is also a lack of attendance at university programs aimed at smoothing the transitioning period, such as orientation [8]. As a result, the students who do not attend these kinds of programs often feel left out and isolated at the commencement of their university life [8], which can lead to academic stress [3]. According to Haktanir et al. [1], the factors that negatively influence resilience among first-year undergraduate nursing students often manifest in the form of academic drop-out, academic and social issues, and mental health problems.

There is therefore a need for this current study, which is part of a doctoral study which seeks to develop and validate a context-specific conceptual framework to improve resilience among first-year undergraduate nursing students. To the knowledge of the researchers, there is no conceptual framework for resilience that is context-specific to the first-year undergraduate nursing students, despite their difficult process of transitioning to the university. Therefore, the aim of this study was to determine factors that influence resilience among first-year undergraduate nursing students at a South African university. The results of this study, together with the findings from related studies [11,12] from a doctoral study referred to earlier were converged to develop and validate a context specific conceptual framework to improve resilience among first-year undergraduate nursing students at a South African university.

## 2. Materials and Methods

A cross-sectional descriptive research design was followed in this study. According to Brink et al. [13], a cross-sectional descriptive design is a type of research design where data are collected from respondents at one specific point in time on a phenomenon of interest to the researcher. This type of research design is widely used in health sciences research.

Data were collected only once from the respondents regarding factors that influence resilience among first-year undergraduate nursing students at a selected South African university. The two main variables in this study were resilience and first-year undergraduate nursing students, with first-year undergraduate nursing students being dependent on various factors influencing their resilience.

### 2.1. Study Setting

The study was conducted in one of South Africa’s universities, which is found in two provinces in the country. The university has three campuses which are approximately 200 km apart from each other. Of the three campuses, only two were accredited to offer the Bachelor of Nursing program at the time of conducting this study. Although the two campuses on which the study was conducted offer an aligned program, the two institutions differ in terms of culture and resources. In addition, one campus is located in a predominantly rural area, whereas the other is located in a semi-urban area.

### 2.2. Population and Sampling

The target population was bona fide first-year undergraduate nursing students (*n* = 156) at a tri-campus South African university, comprising 80 and 76 undergraduate nursing students from campus A and B, respectively. Campus C was not accredited to offer the undergraduate nursing program at the time of conducting this study. Stratified convenient sampling was used to segment and group first-year undergraduate nursing students according to the campuses they came from. This was done to achieve homogeneity of the groups in their heterogenic state due to their campuses of origin [14,15], even though they fall within one school in one university. In each segmentation or stratum, a sample was selected using convenient sampling. A Raosoft sample size calculator, sourced from Las Vegas, United States of America, was used to compute the sample size for the two campuses (*n* = 112). Since the two campuses did not admit the same number of first-year undergraduate nursing students at the time of conducting this study, a proportional allocation of the sample size was applied to ensure a fair and just representativeness of the population [15]. The sample sizes at each campus were computed as shown in Table 1.

Although the sample sizes given in Table 1 represent the smallest sample size needed per campus to attain sample power in this study, a greater number of respondents was welcomed (see Section 3.1).

### 2.3. Recruitment of Participants and Data Collection

Recruitment of participants was carried out by an independent research assistant in a fair and just manner to avoid bias. Subsequently, informed consent was also obtained by an independent research assistant, to avoid prejudice of participants. Data were collected using an online adapted self-administered questionnaire, previously used by Mampane in a similar study in South Africa [16]. Minor adaptations were made to contextualize the questionnaire to the higher education setting, such as replacing words like teacher with lecturer. The questionnaire remained with 29 items as in its original form. Data were collected during August 2023. The link to the data collection tool (https://forms.gle/zgUi8u6Gn736TVHM9, accessed on 1 August 2023), created using Google Forms on Google Drive, was shared through an institutional online teaching-and-learning platform called eFundi. The data collection tool had four sections, namely informed consent, demographic data, adapted questionnaire, and decline to participate. The first section, informed consent, determined access to sections two and three of the questionnaire. That is, respondents who clicked ‘No’ to the question ‘Do you grant consent to participate in this study?’ in the informed consent section were redirected to the ‘Declined to participate’ section without filling in the questionnaire. The type of data that were collected was information relating to the factors that influence resilience among first-year undergraduate nursing students at a selected university. No identifying information was collected, to protect the anonymity and confidentiality of the respondents.

### 2.4. Data Analyses

Data analyses were carried out using SPSS version 28, with the aid of a qualified statistician at the institution of affiliation of the researcher. The statistician completed a standard institution confidentiality agreement form. The Kaiser–Meyer–Olkin Measure (KMO) of sampling adequacy and Bartlett’s Test of Sphericity were used to ascertain that the sample was suitable for exploratory factor analysis (see Section 3—Results). After this, exploratory factor analysis of the questionnaire items was conducted using the principal component analysis extraction method and varimax rotation. Varimax rotation was applied to achieve a simpler structure and enhance interpretability by maximizing the variance of squared loadings within each factor while minimizing cross-loadings. Tables, pie charts, and bar graphs are used to present the results (see Section 3—Results).

### 2.5. Validity and Reliability of the Data Collection Tool

In this section, the validity and reliability of the data collection tool are discussed.

#### 2.5.1. Validity of the Data Collection Tool

According to Maree [15], the validity of a data collection tool is concerned with the extent to which it measures what it is supposed to measure. Therefore, to ensure validity of the data collection tool in this study, both face validity and content validity were ensured. As explained by Maree [15], face validity refers to the extent to which the tool looks valid in the eyes of the experts, whereas content validity refers to the extent to which it measures what it is expected to measure, after thorough scrutiny by experts. The tool was subjected to scrutiny by a team of experts, including a statistician, to ensure its face and content validity.

#### 2.5.2. Reliability of the Data Collection Tool

Reliability of the data collection tool refers to the extent to which the instrument can yield the same results if it were to be used at a different time or administered to different respondents from the same population [15]. Therefore, to ensure that the tool is repeatable and consistent, internal consistency was ensured by running an inter-item Cronbach’s alpha coefficient on SPSS version 28. To indicate internal consistency, the Cronbach’s alpha coefficient had to be above 0.6, with higher values closer to 1 indicating higher reliability [14,15,17]. The data collection tool proved to be reliable, as it had a Cronbach’s alpha coefficient of 0.884 in its original form. The Cronbach’s alpha coefficient was run despite the original data collection tool having a reliability score of 0.82, because the data collection tool was adapted for the context of this study. Following the establishment of internal consistency, the researcher conducted a pilot study with 10% of the population. Since there were no amendments to the tool after the pilot study, the results thereof were included for analysis.

### 2.6. Ethical Considerations

The study was approved by the Health Research Ethics Committee (HREC) of the institution of affiliation and allocated a unique ethics number (NWU-00053-23-A1). Following ethical approval, goodwill permission was sought from the Research Data Gatekeeper Committee of the same institution before interacting with respondents. Upon receipt of goodwill permission (NWU-GK-23-140), permission was sourced from the deputy directors at each campus as the custodians of the students. This is because the school director, who is the rightful mediator, was the co-promoter and could not provide approval. Participation in the study was entirely voluntary, and first-year undergraduate nursing students were recruited by an independent research assistant in a just and fair manner without any coercion. Respondents who showed interest signed the consent form, which was linked to the data collection tool by means of clicking on ‘Agree to participate’. Those who clicked on ‘Do not agree to participate’ were redirected to the last aspect of the data collection tool, which prompted them to either submit without responses or simply exit the browser. First-year undergraduate nursing students were assured that there would be no consequences should they opt not to participate in the study.

## 3. Results

### 3.1. Demographic Data

A total of 123 participants responded to the survey. Sixty-five (52.8%) of the respondents were from the campus B and 58 (47.2%) were from campus A. An overwhelming majority (88%) of the respondents were females, while the remainder (12%) were males. However, no evidence suggests that the gender imbalance influenced the results in one way or another. Furthermore, most (89.4%) of the respondents were aged between 18 and 20 years. This could be attributed to the fact that the university entry exam (Grade 12) in South Africa is written between the ages of 17 and 18 or, in some instances, 19. Ten (8.1%) and 3 (2.4%) of the respondents were in the 21–23 and 24–26 age groups, respectively (see Table 2).

### 3.2. KMO Measure and Bartlett’s Test

Results shown in Table 3, the KMO measure of sampling adequacy was 0.778, suggesting that the data were satisfactorily appropriate for factor analysis. In addition, a highly significant result (χ^2^ (406) = 1504.521, *p* < 0.001) from Bartlett’s test of sphericity supported the applicability of factor analysis on the dataset by showing that the correlation matrix was not an identity matrix.

The Eigenvalues showed that the first factor explained 23.9% of the variance, the second factor 11.2% of the variance, and the third factor 7.5% of the variance, while the fourth factor explained 5.9% of the variance. The fifth, up to and including the eight factors, had Eigenvalues greater than 1 but less than 2. Overall, the eight extracted factors accounted for a cumulative variance of 65.5%. Table 4 lists the factors and factor loadings as well as the item communalities.

Communalities ranged from 0.458 to 0.810 (see Table 4). For ease of interpretation, factor loadings displayed in Table 4 are from the rotated pattern matrix. Eigenvalues of ≥1 were used to interpret the number of factors in the data set.

The frequency and percentage (in brackets) distribution of responses to eight item statements is shown in Table 5. The last three columns describe the descriptive statistics for factor items.

Factor 1 comprises of 8 items as shown in Table 5. A follow-up reliability analysis found factor 1 to have good internal consistency (α = 0.868). Communalities of factor loading 1 show that just above 65% of the respondents agreed with the statement ‘My lecturers make me see that I am good at my work’ most or all of the time. However, approximately 58% indicated that they did not have a lecturer they could talk to. Slightly over 75% of the respondents indicated that the lecturer explained concepts most or all of the time. Almost 90% of the respondents stated that the lecturer worked hard to make them understand concepts. Furthermore, 78% of the respondents stated that the lecturer gave extra examples in class. Approximately 70% and 75% of the respondents indicated that lecturers supported them to aim high as well as think of their bright future, respectively, while almost 65% stated that they had at least one lecturer who encouraged them to do their best. There was an association between the campus and whether the respondent had a lecturer they could talk to ($\chi_{0.05,3}^{2}$ = 18.501, *p* < 0.001). There was also an association between campus and whether the respondent had at least one lecturer who encourages them to do their best ($\chi_{0.05,3}^{2}$ = 11.343, *p* < 0.05).

Factor 2 consists of 6 items, as shown in Table 5 (which indicates the frequency and percentage in brackets), which appeared to measure what it means to students to achieve in class. A follow-up reliability analysis found factor 2 to have acceptable internal consistency (α = 0.712). Communalities of factor loading 2 shows that most (97.6%) of the respondents were of the view that their future and success depended on their hard work. Approximately 96% believed they could do better, while 94% stated that they did all assignments most or all the time. Just below 98% of the respondents indicated that doing well at school was important to them. With respect to being in control of what happened to them, 78% of the respondents indicated that they were in control most or all the time. Almost all (98.4%) of the respondents stated that their future was in their hands. There was an association between ‘My future and success depend on my hard work’ and campus ($\chi_{0.05,3}^{2}$ = 7.998, *p* < 0.05), age group ($\chi_{0.05,3}^{2}$ = 25.052, *p* < 0.001) and gender ($\chi_{0.05,3}^{2}$ = 9.204, *p* < 0.05). There was also an association between the respondents’ belief to do better and campus ($\chi_{0.05,3}^{2}$ = 8.070, *p* < 0.05). The importance of doing well at school was associated with the campus ($\chi_{0.05,3}^{2}$ = 8.671, *p* < 0.05) and age group ($\chi_{0.05,3}^{2}$ = 16.808, *p* < 0.01). Whether a respondent was in control of what happens to them was associated with the campus ($\chi_{0.05,3}^{2}$ = 16.283, *p* < 0.001).

Factor 3 consists of 3 items, as shown in Table 5, which appeared to measure the influence of support from peers or mentors in relation to students’ resilience. A follow-up reliability analysis found factor 3 to have acceptable internal consistency (α = 0.742). Regarding the statement ‘I know someone at the university who cares about me’ in Table 5, most respondents (44.7%) said it was true most of the time, with almost 27% believing it was true all the time. Similarly, for the statement ‘I know someone at the university I can talk to’, the majority (40.7%) said it was true most of the time, with approximately 28% saying it was true all the time. These findings indicate that a sizeable proportion of respondents believe they have helpful contacts that are approachable within the campus community. Furthermore, when it came to the statement ‘I have good talents’, a sizeable majority (52%) said it was true most of the time, with 30% believing it was true all the time. There was an association between the respondents’ assertion of having good talents and age group ($\chi_{0.05,3}^{2}$ = 13.030, *p* < 0.05). This demonstrates that respondents are confident in their abilities or strengths, reflecting a favourable self-perception in the academic setting. Overall, the findings show that students in the university setting have a generally favourable attitude regarding interpersonal connections, support networks, and self-esteem.

Factor 4 comprises 4 items, as shown in Table 5, which appeared to measure students’ determination to achieve their goals. A follow-up reliability analysis found factor 4 to have acceptable internal consistency (α = 0.736). The information in factor 4 provides a convincing peek into people’s attitudes and beliefs about resilience, problem-solving, and optimism. Notably, most responders indicated strong positive feelings about perseverance and optimism. For example, when it comes to believing in a brighter future, an overwhelming 75.6% said they feel things would eventually improve for them, with another 22.8% saying they thought this most of the time. This largely positive view shows that the individuals had a resilient mindset. There was an association between the respondents’ belief in a brighter future one day and campus ($\chi_{0.05,3}^{2}$ = 8.654, *p* < 0.05). Similarly, when it came to problem-solving tactics, 61.8% reported adopting a variety of methods to solve difficult problems most of the time, emphasizing adaptation and flexibility in handling challenges. Furthermore, nearly half of the respondents (48.8%) stated that they do not allow people to obstruct their efforts, while 44.7% stated that they do so most of the time. There was also an association between the respondents’ assertion that they do not allow people to stop them from trying to do their best and gender ($\chi_{0.05,3}^{2}$ = 7.948, *p* < 0.05). In terms of perseverance, an encouraging 49.6% said they never give up, with a further 42.3% saying they persevere most of the time. Overall, these findings indicate a prevalent attitude of resilience, determination, and a constructive view among those surveyed, emphasizing their tendency to persevere in the face of adversity while maintaining an optimistic outlook on their future possibilities.

Factor 5 comprises 3 items, as shown in Table 5, which appeared to measure the support students receive from an adult figure. A follow-up reliability analysis found factor 5 to have acceptable internal consistency (α = 0.778). Communalities of factor loading 5 shows that 83% of respondents had an adult to talk to most or all the time, compared to 17% who said they did not have an adult to talk to. Having an adult to talk to had no association with the campus, gender, or age group. Furthermore, the majority (86%) of respondents answered that they have an adult who listens most or all of the time. In comparison, 3% and about 11% did not have such an adult all or most of the time, respectively. There was no association found between having an adult who listens and being on a certain campus, age group, or gender. In terms of feeling safe and loved at home, nearly 95% of respondents said they felt so most or all of the time. Less than 1% and 4%, respectively, did not feel safe or loved at home all or most of the time. Gender was associated with feelings of safety and being loved at home ($\chi_{0.05,3}^{2}$ = 8.973, *p* < 0.05).

Factor 6 consists of 3 items, as shown in Table 5, which appeared to measure students’ level of self-determination to achieve and inspiration from others. A follow-up reliability analysis found factor 6 to have poor internal consistency (α = 0.596). Items in factor 6 indicate that a considerable proportion of participants showed a favourable inclination for emulation and inspiration, with 46.3% stating that they know a good person whose behaviour serves as an example to them, and a further 38.2% stating that this was the case most of the time. When it came to class attendance and dedication, a substantial majority (54.5%) expressed a strong preference for not being absent from class, with 38.2% indicating this preference most of the time. This reflects a general sense of dedication and importance placed on regular attendance among respondents. Furthermore, the data reveal a strong feeling of determination and persistence, with 47.2% of people saying they never give up trying, and another 42.3% saying that this is the case most of the time. Overall, the data indicate a favourable trend toward role model recognition, attendance commitment, and a resilient attitude among those surveyed, indicating a proactive and motivated approach to personal improvement and goal attainment.

### 3.3. Descriptive Statistics for Items in Each Factor

For almost all the factors, item mean scores were above 2.50, indicating that the respondents leaned towards agreement with the statements. For items with means of at least 3.00, respondents indicated that the items were mostly true, with occasional deviations. This suggests that respondents more consistently endorsed the statements. Only a single item, ‘There is one lecturer that I can talk to who listens to me and encourages me to do my best’, had a mean below 2.50. This suggest that respondents did not endorse this statement.

Statistics in the last column provide insight into the average responses and variability for each factor in the study. Factors 2, 4, 5, and 6 have higher means compared to factors 1 and 3, indicating stronger agreement with the statements associated with these factors. Factors 1 and 3 have lower means, suggesting less agreement. The standard deviations show the degree of variability in responses within each factor.

## 4. Discussion

The purpose of this study was to determine the factors that influence resilience among first-year undergraduate nursing students. Question items were grouped together through data analysis to reveal six factors that were highlighted as influencing resilience among the first-year undergraduate nursing students: lecturer support, parental support, academic achievement, peer and mentor support, optimism about the future, and a feeling of determination. These factors are discussed further in this section.

Communality factor loading 1 was termed lecturer support, which was measured using eight data sets. The respondents were in general agreement with the item statements that described lecturer support, implying that this can be considered as a factor that positively influences resilience among first-year undergraduate nursing students. Since the transition to university is demanding and stressful in nature for first-year undergraduate nursing students, a considerable amount of support from lecturers goes a long way in ensuring that students acclimatize well to the university. The result of this study concurs with a study conducted by Warshawski [18], which cites that support from lecturers through individualized or intimate group sessions can improve students’ resilience. The same author adds that in instances where lecturer support is lacking, students usually lack motivation and end up demoralized. Furthermore, a study conducted by Lekan et al. [2] cited that one role of a nurse educator is to assist students to become more resilient. This further affirms the pivotal role played by lecturer support in influencing resilience among first-year undergraduate nursing students.

Question items in communality factor loading 2 were grouped together to reveal the second factor that influences resilience among first-year undergraduate nursing students: academic achievement. The results of this study proved that with academic success comes motivation and thus resilience, with most students agreeing with question items that described the academic achievement factor. Similarly, a study conducted by Lekan et al. [2] reported a directly proportional relationship between resilience and academic success. Unlike the current study, Lekan et al. [2] associated being more resilient with the likelihood of academic success. Furthermore, a study conducted by Warshawski [18] also cited that resilience has been found to significantly influence academic success. Therefore, whether resilience influences academic success or vice versa, academic success is a factor that can motivate students and ultimately improve their resilience.

Three question items (factor loading 3) were grouped to describe the third factor, which is the value of support received from peers and mentors. The results showed that, first-year undergraduate nursing students value peer and mentor support as it plays a critical role in improving their resilience. The results are consistent with those of a study conducted by Lanz [19], which reported that undergraduate nursing students proposed reduced lecture times and increased group interaction as a strategy to improve their resilience. Furthermore, a study conducted by Warshawski [18] during the COVID-19 pandemic elucidated that undergraduate nursing students found it hard to cope with the transition to university, thus further ascertaining that peer and mentor support, as part of social support, is critical to ensure resilience among first-year undergraduate nursing students, as purported in this study. In addition, Warshawski [18] further alluded that the undergraduate nursing students explained the need for an academic adviser to improve their studies. Similar to the results in the current study, the majority (68.3% and 71.5%) of the respondents agreed with the statements ‘I know someone to talk to at the university’ and ‘I know someone who cares about me at the university’, respectively. As Taarega et al. [20] and Hughes et al. [21] explain, having someone near a first-year undergraduate nursing student to give support and advice when needed is vital to improve their resilience, especially when faced with academic challenges. On the contrary, realisation of peer and mentor support seems abstract within the context of South African Higher Education due to the amount of academic workload that first-year undergraduate nursing students have [11]. First-year undergraduate nursing within the South African Higher Education context endures long hours of class, group works and clinical placement which leaves little to no time for social interactions, which is a platform for peer and mentor support [11].

Second from last, two sets of question items were grouped to create two factors, namely optimism about the future and a feeling of self-determination, which are discussed together because they are closely related. The results showed that respondents agreed with question items describing these two factors, thus implying that with optimism and self-determination, resilience is somewhat guaranteed. Similar results were found in a study conducted by Porter [22], who stated that optimism empowers students with problem-solving skills, which allows them to defeat potential adversities. In addition, Smith and Gregg [23] attribute optimism to be a critical factor in the process of growing. In this study, optimism about the future was seen to be a factor influencing resilience among first-year undergraduate nursing students. The results further showed that resilience among these students can be improved if they have an increased feeling of self-determination. Previous studies have reported similar results [21]. According to Hughes et al. [21], one of the key aspects that smooths the transition to the university for first-year undergraduate nursing students is facing the demands of post-secondary schooling. This can only be possible when students start to display a feeling of self-determination.

Question items in communality factor loading 5 were combined to assess the support the students get from parents or parental figures, and how that influences their resilience. The results show that parental support plays a crucial role in influencing the resilience of first-year undergraduate nursing students at the university. However, parental support referred to in this study does not necessarily have to be physical nurturing, being dependent on, or residing within the same household with parents, but was measured through having an adult to talk to or someone who cares about a student, as depicted in the question items in Table 5. This is because the entire period of being at university can be attributed to the transition to independence [11]. The results of this study are supported by Lee and Fletcher [24], who reported that parental support is vital and beneficial for adjustment of first-year undergraduate nursing students in institutions of higher learning. Similarly, McCulloh [25] reported that parental support is perceived by first-year undergraduate nursing students as an effective method to foster students’ retention at the university. The same authors cite that parental support for first-year undergraduate nursing students can be associated with memorable and encouraging messages from parents.

### 4.1. Limitations

The study used a cross-sectional descriptive design to collect data. A longitudinal study would have been more comprehensive, as it would show the improvements in resilience among undergraduate nursing students, tracing this from first year to higher levels. In addition, few participants were able to participate due to technical challenges with the online link, since the entire method of data collection was online. Therefore, for future studies the researcher might consider a longitudinal study at postdoctoral level. Furthermore, the results presented are specific to the context in which this study was conducted. As a result, caution should be exercised when generalizing the results.

### 4.2. Recommendations

The authors recommend further research on a similar phenomenon using other designs, such as intervention studies, appreciative inquiry, and/or longitudinal studies. In addition, the authors recommend that nursing education institutions consider the factors presented in this study during the orientation of first-year undergraduate nursing students.

## 5. Conclusions

Resilience is a vital trait needed to ensure the academic success of the first-year undergraduate nursing students, ensuring retention in the programme and ultimately course completion. This study showed that resilience can be dependent on factors such as lecturer support, parental support, academic achievement, peer and mentor support, optimism about the future and self-determination.

## Figures and Tables

**Table 1:** Sample sizes from the two campuses where study was conducted.

Description	Campus A	Campus B
Population in numbers	80 first-year undergraduates	76 first-year undergraduates
Proportional allocation in %	(80 ÷ 156) × 100 = 51%	(76 ÷ 156) × 100 = 49%
Proportional sample size	80 ÷ 156 = 0.51282051	76 ÷ 156 = 0.48717949
Sample size in numbers	0.51282051 × 112 = 57	0.48717949 × 112 = 55

**Table 2:** Demographic characteristics.

Characteristic	Result	No.	%
Total number of participants	Campus A	*n* = 58	47.2
	Campus B	*n* = 65	52.8
Gender	Male	*n* = 15	12.0
	Female	*n* = 108	88.0
Age group (years)	18 to 20	*n* = 110	89.4
	21 to 23	*n* = 10	8.1
	24 to 24	*n* = 3	2.4

**Table 3:** Sampling adequacy table.

Kaiser–Meyer–Olkin Measure of Sampling Adequacy		0.778
Bartlett’s Test of Sphericity	Approx. χ^2^	1504.521
	df	406
	Significance	<0.001

**Table 4:** Factor loadings and communalities.

Factors	1	2	3	4	5	6	Communalities
My lecturers support me to think of my bright future	**0.814**						**0.711**
My lecturers support me to aim high	**0.809**						**0.781**
There is at least one lecturer who encourages me to do my best	**0.766**						**0.691**
My lecturers make me see that am good with my work	**0.675**						**0.606**
There is at least one lecturer that I can talk to who listens to me and encourages me to do my best	**0.671**						**0.603**
Lecturers explain a lot in class	**0.647**						**0.593**
My lecturer works hard to help me understand my work better	**0.588**						**0.545**
Lecturer gives extra examples in class	**0.586**						**0.645**
My future and success depend on my hard work		**0.718**					**0.698**
I believe that I can do better		**0.678**					**0.588**
I make sure that I do all my assessments		**0.675**					**0.577**
Doing well at school is very important to me		**0.673**					**0.537**
I am in control of what happens to me		**0.536**					**0.547**
My future is in my hands		**0.404**					**0.458**
I know someone at the university who cares about me			**0.856**				**0.803**
I know someone at the university that I can talk to			**0.825**				**0.763**
I believe I have good talents			**0.402**				**0.601**
I believe that one day things will be better for me				**0.794**			**0.716**
Even when my problems are just too much, I do not give up trying to make it work				**0.707**			**0.668**
I use different ways to work out a difficult problem				**0.551**			**0.566**
I do not allow people to stop me from trying to do my best in my work				**0.452**			**0.499**
I have an adult to talk to at home					**0.875**		**0.810**
I have an adult who listens to me					**0.869**		**0.805**
I feel safe and loved at home					**0.577**		**0.740**
I know a good person whose behaviour is an example to me						**0.764**	**0.710**
Even when I don’t understand in class, I don’t give up trying						**0.698**	**0.623**
I do not like to be absent from classes						**0.487**	**0.606**

Note: Factor loadings are in bold. Extraction method: principal component analysis. Rotation method: varimax rotation.

**Table 5:** Frequency distribution and descriptive statistics of communalities of factor loadings 1 to 5.

Item	Untrue All the Time	Untrue Most of the Time	True Most of the Time	True All the Time	Mean	SD	Factor Mean (SD)
**Communalities of factor loading 1 (lecturer support)**							
My lecturers make me see that I am good with my work	12 (9.8)	29 (23.6)	57 (46.3)	25 (20.3)	2.77	0.89	2.87 (0.62)
There is at least one lecturer that I can talk to who listens to me and encourages me to do my best	36 (29.3)	35 (28.5)	37 (30.1)	15 (12.2)	2.25	1.01	
The lecturer explains a lot in class	4 (3.3)	26 (21.1)	56 (45.5)	37 (30.1)	3.02	0.80	
Lecturers works hard to make me understand	1 (0.8)	13 (10.6)	65 (52.8)	44 (35.8)	3.24	0.67	
Lecturers give extra examples in class	4 (3.3)	23 (18.7)	57 (46.3)	39 (31.7)	3.07	0.80	
My lecturers support me to aim high	10 (8.1)	27 (22.0)	60 (48.8)	26 (21.1)	2.83	0.86	
My lecturers support me to think of my bright future	8 (6.5)	23 (18.7)	55 (44.7)	37 (30.1)	2.98	0.87	
There is at least one lecturer who encourages me to do my best	13 (10.6)	31 (25.2)	51 (41.4)	28 (22.8)	2.76	0.92	
**Communalities of factor loading 2 (academic achievement)**							
My future and success depend on my hard work	1 (0.8)	2 (1.6)	12 (9.8)	108 (87.8)	3.85	0.46	3.62 (0.38)
I believe I can do better	0 (0.0)	5 (4.1)	30 (24.4)	88 (71.5)	3.67	0.55	
I make sure that I do all my assignments	7 (5.7)	0 (0.0)	48 (39.0)	68 (55.3)	3.50	0.61	
Doing well in school is important to me	0 (0.0)	3 (2.5)	24 (19.5)	96 (78.0)	3.76	0.49	
I am in control of what happens to me	5 (4.1)	22 (17.9)	48 (39.0)	48 (39.0)	3.13	0.85	
My future is in my hands	2 (1.6)	0 (0.0)	18 (14.6)	103 (83.7)	3.80	0.51	
**Communalities of factor loading 3 (peer and mentor support)**							
I know someone at the university who cares about me	17 (13.8)	18 (14.6)	55 (44.7)	33 (26.8)	2.85	0.98	2.92 (0.74)
I know someone at the university that I can talk to	15 (12.2)	24 (19.5)	50 (40.7)	34 (27.6)	2.84	0.97	
I believe I have good talents	4 (3.3)	18 (14.6)	64 (52.0)	37 (30.1)	3.09	0.76	
**Communalities of factor loading 4 (optimism about the future)**							
I believe that one day things will be better for me	2 (1.6)	0 (0.0)	28 (22.8)	93 (75.6)	3.72	0.55	3.47 (0.44)
I use different ways to work out a difficult problem	4 (3.3)	7 (5.7)	76 (61.8)	36 (29.3)	3.17	0.67	
Even when my problems are just too much, I do not give up trying to make it work	2 (1.6)	8 (6.5)	52 (42.3)	61 (49.6)	3.40	0.69	
I do not allow people to stop me from trying to do my best	2 (1.6)	6 (4.9)	55 (44.7)	60 (48.8)	3.41	0.66	
**Communalities of factor loading 5 (parental support)**							
I have an adult to talk to at home	4 (3.3)	17 (13.8)	51 (41.5)	51 (41.5)	3.21	0.80	3.39 (0.61)
I have an adult who listens to me	4 (3.3)	13 (10.6)	52 (42.3)	54 (43.9)	3.27	0.78	
I feel safe and loved at home	1 (0.8)	5 (4.1)	24 (19.5)	93 (75.6)	3.70	0.59	
**Communalities of factor loading 6 (a feeling of self-determination)**							
I know a good person whose behaviour is an example to me	8 (6.5)	11 (8.9)	47 (38.2)	57 (46.3)	3.24	0.87	3.36 (0.55)
Even when I do not understand in class, I do not give up trying	2 (1.6)	11 (8.9)	52 (42.3)	58 (47.2)	3.40	0.69	
I do not like to be absent from class	0 (0.0)	9 (7.3)	47 (38.2)	67 (54.5)	3.47	0.63	

## Data Availability

Data that were used to support the reported results can be obtained from the corresponding author of this study upon a reasonable request.

## Public Involvement Statement

No public involvement in any aspect of this research.

## Guidelines and Standards Statement

This manuscript was drafted against the STROBE reporting guideline for cross-sectional descriptive research. A complete list of reporting guidelines can be accessed via the equator network: https://www.equator-network.org/, accessed on 1 August 2023.
